# Collectanea: Pathology and Practice

**Published:** 1835-04-01

**Authors:** 


### COLLECTANEA.

### PATHOLOGY AND PRACTICE.

### DR. GÖLIS'S TREATMENT OF THE DISEASES OF CHILDREN.

We are indebted for the following long but valuable article to the Gazette Médicale, of the 25th October 1834, and the 31st January 1835. It is an account of the practice of that distinguished physician, Dr. Gölis, of Vienna, in the diseases of children, and was drawn up by Dr. Brosius, his pupil.

*Inflammatory Diseases.* According to Dr. Gölis, two thirds of the diseases of childhood are inflammatory; stimulants must therefore be administered but sparingly, and we must often confine ourselves, especially when the case is doubtful, to mild and gentle treatment. Febrile diseases observe a regular type, and the exacerbations recur at fixed hours; it is only occasionally that they are hastened or retarded a little. Inflammatory fevers are marked by a peculiar brilliancy of the eyes. If fevers of this kind are complicated with worms, they are sure to be attended by anomalous symptoms.

*Pneumonia.* The pneumonia of children is recognized chiefly by the pain produced by respiration and coughing. The following is Dr. Gölis's usual prescription in this and other inflammatory diseases. ℞. Inf. Glycyrrhizæ rad: Decoct. Sem. Lin. *aa* ℥ij.; Potassæ Nitratis ℈j.; Oxymel Simpl. ℥iss.; M. sumat coch. j. min. omni horâ. (For a child about two years old.)

This mixture of an infusion of liquorice root and a decoction of linseed, is the vehicle for the majority of potions prescribed at the children's hospital at Vienna. In private practice Dr. Gölis uses in its stead a decoction of the root of mallow or saloop. When resolution has taken place, the acetate of ammonia is substituted for the nitrate of potash, a drachm being added to the above mixture.

In the pneumonia of rachitic children, Dr. Gölis has a great predilection for the acetate of ammonia, because it stimulates a little; for in cases of this kind there is relaxation of the bronchi and accumulation of mucus. What Schöffer, in his treatise on the diseases of children, calls *pulmonary paralysis*, is, according to Dr. Gölis, a real bronchitis, and should not be treated with musk, but with leeches and blisters.

*Angina Gutturalis.* (Cynanche tonsillaris.) Dr. Gölis shows great dexterity in opening children's mouths, to look at the throat; he introduces his little finger into the mouth, and presses it on the base of the tongue. This causes an attempt at vomiting, opening the throat, and enabling him to satisfy himself as to the existence of an angina gutturalis. This disease is often accompanied by retching: when it is situated in the œsophagus, children keep their neck stiff.

We must never omit to look at the cavity of the throat, when catarrh prevails epidemically, or we shall run the risk of overlooking many an angina. If this angina is accompanied by fever, nitre is to be given, as in pneumonia. If there is no fever, the acetate of ammonia is to be given. Besides these internal remedies, the neck is to be warmly wrapped up, and covered with bags of medicinal herbs.

*Angina Serosa.* This disease consists in swelling without redness, and is treated with the acetate of ammonia.

*Angina Membranosa*, or Croup. When the appearance of croup exactly coincides with that of the eruption of measles, it is not to be feared; but it is dangerous if it appears while measles is running its course, or afterwards. When a child is affected with croup, he must not be suffered to sleep for more than half an hour at a time; he must be kept awake by continually giving him lukewarm drink. When calomel produces liquid stools, its use must be suspended for a short time; for, if persisted in, it might easily cause an enteritis. The angina would disappear, but the child would inevitably be lost. We are not to use calomel with too much diligence, if the child is of a scrofulous habit, for this medicine might easily change a simple attack into a decided scrofulous affection. We must also be aware of administering musk too soon against the remains of a spasmodic cough, because if the slightest inflammation should still be present, this drug might cause a relapse. It is better to persist in the use of antiphlogistic remedies. The means employed by Dr. Gölis in croup are those adopted by all good practitioners: leeches, calomel, which is sometimes administered in the dose of a grain every hour, with sugar, besides frictions with a mixture of mercurial and mallow ointment on the neck and upper part of the chest; nitre in the intervals; emetics when the breathing is stertorous; and lastly blisters, which, when employed in time, are, according to Dr. Gölis, the most powerful method of preventing membranous exsudation.

The emetic which Dr. Gölis generally prefers for children, consists of Antim. Tart. with a few grains of Ammon. Mur. dissolved in distilled water. He considers the sulphuret of potash as inefficacious, and as difficult to administer from the disgust which it excites, and therefore one with which we ought not to waste precious time.

*Inflammation of the Cavity of the Mouth, or Stomatitis.* In this affection the acetate of ammonia is given internally. If there are small ulcers, they are to be touched with the following mixture: ℞. Mellis Rosæ ℥iss.; Mucil. Cydon. ℥j.. M. Borax is too stimulating.

*Difficult Dentition.* This affection is easily recognized by the rose colour of the gums, which enlarge at the spot where the tooth is to come through. If dentition is very painful, it is often accompanied by an eruption, and in plethoric children, who have porriginous crusts upon their head, the porrigo sometimes increases suddenly.

*Inflammation of the Spinal Marrow, or Myelitis.* When a child presents the following symptoms, we may be sure that it labours under myelitis. The body is quite straight from head to foot; the arms are strongly fixed against the chest; the fore-arms move but feebly in the elbow joint, but rather more in pronation and supination; the hand can sometimes be carried up to the chest, but rarely as far as the mouth. The lower extremities are firmly applied to one another in their whole length, and if we endeavour to separate them, the child cries. It also cries out loudly if the position of its body is changed, as, for example, if any one takes hold of it by the shoulder. The disease is also characterized by a great tendency to diarrhœa. Such are the symptoms of myelitis, before the inflammation attacks the brain, but when that is implicated, convulsions appear, and the diarrhœa ceases.

The treatment is antiphlogistic.

*Acute Hydrocephalus.* If a child cries constantly from the day of its birth, if it eats a great deal, and the stools are greenish, if it lies quite straight in bed, and throws its head back, burying the occiput in the pillow, we may be sure that hydrocephalus will occur. When Dr. Gölis is apprehensive of this disease, he inquires if the children are cross, if they have become indifferent to what they most liked before, if they sigh frequently, and sometimes vomit, and if they often stare in a fixed and dreamy manner. A nasal utterance is also to be reckoned among the pathognomonic signs, and also the fact of the child's attempting to catch hold of things on one side. In a word, the look, the physiognomy, the gestures of a child threatened with hydrocephalus, are all full of meaning to the experienced practitioner.

A symptom never wanting in acute hydrocephalus, is a speedy diminution of the size of the abdomen. In children thus affected, the belly, however large, becomes flat during the inflammatory stage; and during the period of coma, this is the most certain diagnostic mark between acute hydrocephalus and typhus, in which the belly is most frequently tympanitic. However large the belly may have been before the disease, the bowels are so much retracted towards the vertebral column, that one might imagine that they had entirely disappeared. The most frequent causes are contusions of the head, concussion of the brain through falls, blows, &c.

When the disease is merely suspected, calomel and tartar emetic ointment may be employed by way of precaution. Calomel is generally the chief remedy in this disease; it is given in the dose of half a grain every hour or every two hours, or a grain every three hours, according to circumstances, such as the existence of constipation.

The application of cold lotion to the head, and the use of nitre in the inflammatory stage, may be continued without inconvenience when effusion has taken place; because (says Dr. Gölis), when this period has once arrived, nothing can do much harm. He says, that cold effusions and the application of sulphuric ether to the head, which have been recommended by Formey in the stage of effusion, are of no use.

When acute hydrocephalus is accompanied by a watery diarrhœa, effusion does not easily take place. A favourable issue may also be anticipated if the child recovers the power of moving his head, and can turn it in every direction. It is well also, to find the pulse losing its usual slowness, and becoming again regular and febrile: this is a sign of the commencement of absorption.

Diseases of the Chest.—*Simple Cough.* When there is merely a simple cough, Dr. Gölis gives the infusion of liquorice root with the emollient decoction already mentioned, adding a few drops of Sydenham's laudanum and simple oxymel. If the cough becomes thick, instead of simple oxymel, he uses oxymel of squills. Thus, for a child two years old he prescribes ℞. Inf. Glycyrrhizæ rad.; Decoct. Lini Sem. (vel Althææ) *aa* ℥j.; Laudani Sydenhami gtt. ij.; Oxymellis Simpl. vel Scillæ ʒij. M. sit dosis coch. j. min.

When the cough is obstinate, or there is a neglected catarrh, Dr. Gölis strongly recommends dulcamara in the following form:

Inf. Glycyrr. et Decoct. emoll. ℥iij.; Extr. Dulcamaræ gr. x.; Laudani Sydenhami gtt. ij.; Oxym. Simpl. ʒiij. M. The dose is to be a teaspoonful for a child two years old.

In the case of a child of four or five, who had an obstinate cough with fetid expectoration, Dr. Gölis gave a powder composed of equal parts of liquorice and vegetable charcoal, in the dose of a teaspoonful, several times a day, and prescribed in addition a ptisan of lichen.

*Convulsive Cough.—Hooping Cough.* Dr. Gölis always treats hooping cough according to the prevailing character of the disease, and he teaches that we cannot be successful in the treatment, unless we know if the epidemic is of an inflammatory, catarrhal, or purely nervous kind. When there is no fever, and the disease is nervous, he prescribes ℞. Moschi, gr. iij.; Extr. Opii. aquosi gr. ss.; Acac. gumm. pulv. ℈j.; Sacchari albi, ʒij. M. and divide in pulv. vj.; sumat j. tertiis horis.

Dr. Gölis also extols belladonna in hooping cough; but this remedy requires for its employment, that the disease should be purely spasmodic, and not inflammatory, or catarrhal. When belladonna is indicated, he prefers the root in the following form: ℞. Bellad. rad. gr. j.; Extr. Opii aquosi, gr. ss.; Sacchari albi, gr. lxxx. M. ft. pulv. viij.

One of these powders is to be taken morning and evening, or every three hours, according to circumstances, until the face becomes more animated.

At the same time he prescribes the following embrocation to be rubbed upon the epigastrium: ℞. Tr. Aromat. ℥ss.: Laudani Syden. ʒss.: or the following mixture, which is spread upon soft leather, and applied to the region of the stomach: ℞. Electuar. Anodyni: Bellad. rad. pulv.: Laud. Syden. *aa* ℈j.: Gumm. Arab. ℥j.

A ptisan is to be employed, made of dulcamara, and the roots of liquorice and mallow.

In an epidemic hooping cough which occurred at Vienna in 1816, Dover's powder combined with emollients produced the most fortunate results, when administered as follows: ℞. Inf. Glycyrr. rad.; Decoct. Emoll. *aa* ʒiss.; Potassæ Nitr. gr. x.; Pulv. Doveri, gr. j. vel. ij.; Laud. Syden. gtt. ij.; Syr. Simpl. j. A teaspoonful of this mixture was given every hour to a child two years old.

Frictions with tartar emetic ointment torment children, and, for the most part, uselessly; and even when they lessen the hooping cough, the convalescence of the little patients is always protracted, principally because frictions of this kind destroy the appetite for a long time.

If there is no fever during the second stage of the disease, children bear cold drinks very well; otherwise, all their drink must be lukewarm.

Improvement begins as soon as the intervals between the fits of hooping increase. The disease is often succeeded by an obstinate cough, during which the children throw up sputa, as if they were phthisical. They should then take a decoction of saloop, fourteen ounces being made from fifteen grains, and an ounce of syrup of poppies added; and an infusion made of one ounce of mallow, and half an ounce of dulcamara and liquorice root.

*Periodic Asthma of Children.* In this affection Dr. Gölis prescribes: ℞. Moschi, gr. ij.; Mucil. Acac., ʒij.; Aq. Tiliæ flor., ℥ij.; Ammon. Succin. gtt., ij.; Syrupi Anthem., ℥ss.; M. A teaspoonful is to be given every hour to a child of a year old.

At the same time he orders warm baths, to which pearl-ash and an infusion of chamomile are added. The child remains a quarter or half an hour in the water, and is then immediately wrapped in warm linen and put to bed; the whole being done to promote diaphoresis. The potion is also given: ℞. Valerianæ rad., ʒss.; Fiat Inf., ℥iij. tum adde; Ammon. Succin. gtt. viij. ad x.; Syr. Anthem., ℥ss. At a later stage, blisters are applied to the chest.

*Periodic Suffocation.* Fits of suffocation are sometimes observed in children; they become quite blue, and out of breath. These fits return, but not at regular intervals. According to the experience of Dr. Gölis, they may be caused by an effusion of water in the brain; or they may indicate the first stage of chronic hydrocephalus. If the disease is accompanied by fever, this must be combated first of all; and it will then be well to administer small doses of calomel alternately with the following powder; ℞. Valerianæ pulv. gr. v.; Calcis carb.; Sacchari albi *aa* ℈ss.; M. ut ft. pulv. t. d. sumendus. Baths, impregnated with chamomile and pearl-ash, are also to be ordered, as in the case of periodic asthma.

*Palpitation of the Heart.* Dr. Gölis treats this as a separate disease, and orders a few drops of the following mixture to be taken three times a day: ℞. Tr. Digit., ʒiss.; Ammon. Succin., ʒss.; Laudan. Syden., ℈j.; M.

*Hemoptysis.* In this disease Dr. Gölis extols an emulsion of gum combined with decoction of digitalis, particularly when detraction of blood in any shape is contra-indicated by a state of cachexia. This cures the hemoptysis without leaving any induration behind as astringents do, in consequence of which a little hard cough usually remains.

DISEASES OF THE ABDOMEN. *Diarrhœa.* When diarrhœa in children is accompanied by pain in the belly, we may always admit the existence of a sub-inflammatory state. Diluents alone must then be administered. In simple ordinary diarrhœa, a mixture is prescribed, consisting of two ounces of a decoction of mallow and saloop, and two drops of Sydenham's laudanum. The dose is a teaspoonful every hour or every two hours.

In catarrhal diarrhœa Dr. Gölis prescribes as follows: ℞. Decoct. Althææ, ℥iij; Extr. Dulcam. gr. iij.; Laud. Sydenh. gtt. ij.; Syr. Papav. ℥ss.; M. sit dosis coch. j. min. omni horâ.

If a diarrhœa of this kind lasts long, camphor in small doses becomes an excellent remedy; it produces a peculiar and comfortable sensation of warmth in the stomach. Thus he prescribes, ℞. Camph. solutæ gr. ss.; Decoct. Emoll. ℥j. ad ℥ij.; Laudani gtt. j. ad ij.; M. sit dosis coch. j. min. omni horâ vel omni 2 â h.

When a tonic is required in chronic diarrhœa, he uses calumba; but there must be no fever, and the intestines must not have been despoiled of their mucus by violent alvine evacuations. Half a drachm of calumba and ten grains of saloop are to be boiled for a quarter of an hour, so that the strained decoction may amount to three ounces; half an ounce of syrup of chamomile is then to be added, and the dose is to be a teaspoonful every hour. Or a mixture may be employed consisting of two ounces of a decoction made with eight grains of saloop, eight or ten grains of powdered calumba, two drops of laudanum, and half an ounce of syrup of poppies. To be given like the last mixture, shaking the bottle every time.

If atony already exists, fifteen drops of Hoffmann's elixir (Sp. æther. Sulph. C.) may be substituted for the calumba.

In a chronic diarrhœa following a mucous fever Dr. Gölis prescribed ℞. Arnicæ rad. ʒss.; Calumbæ rad. ℈ij. Macera per horæ quartam partem, et post colationem restent unciæ quatuor, tum adde Laudani gtt. ij.; Syr. Menth. ℥ss.; the dose to be a teaspoonful for a child three years old. When diarrhœa causes a flaccidity of the intestines, Dr. Gölis prescribes, Aq. destill. ceras. nigr. ℥iij.; Extr. Taraxaci ʒij.; Extr. Rhei ʒss.; Ammon. Muriatis gr. vi.; Syrupi Menthæ ℥j. M. sit dosis coch. j. min. He orders, moreover, the whole abdomen to be rubbed with the Ung. nervinum, made stronger by the addition of camphor.

When a chronic diarrhœa is complicated with worms, Gölis prescribes as follows: ℞. Valerianæ rad. ʒss.; Calumbæ rad. ʒj. Aquæ q. s. ut post colationem infusi, restent ℥v., quibus adde, Camph. solutæ gr. j.; Laudani gtt. ij. vel iij.; Syr. Aurant. ℥ss. The dose to be a teaspoonful for a child four years old.

In rachitic children diarrhœa produces prolapsus of the rectum.

*The Cholera of Children.* When this disease is of an inflammatory kind, it is very serious; gangrene is apt to occur, and the child is lost. A sinapism applied to the belly until the skin begins to grow red may sometimes save the little patients. In ordinary cholera, Dr. Gölis gives the following medicines internally: ℞. Inf. Glycyrr. c. Decoct. Emoll. ℥ij.; Laudani gtt. ij; Sp. Æther. Sulph. Comp. gtt. vi.; Syr. Papav. ʒij. And the following ointment to be rubbed upon the epigastrium: ℞. Ung. nervini ℥ss.; Camph. ℈ss.; Opii gr. ij.

The ether, however, is not always advisable at the beginning of the disease, and Dr. Gölis then prescribes a potion consisting of a decoction of three ounces made with eight grains of saloop, with two drops of laudanum, and half an ounce of syrup of poppies.

*Vomiting.* Dr. Gölis treats cases of obstinate vomiting in the same manner as cholera.

*Constipation.* The following remedies are prescribed against ordinary constipation: ℞. Inf. Glycyrr. et Decoct. emoll. ℥iij.; Magnesiæ Sulph. ʒj. ad ʒij.; Syr. ℥ss. Or else, ℞. Inf. Fœniculi, Aq. Fœniculi *aa* ℥ij.; Tr. Rhei aquosæ ʒij.; a teaspoonful to be taken every hour.

*Colic.* Colic may be easily recognized in children; they are restless, cry constantly, stamp on the ground, draw the thighs up to the belly, have twitchings of the face during sleep, and awake crying. When we examine a child whom we suppose to be suffering from colic, we must not neglect to see if its sufferings proceed from some external cause, such as the pinching of some part of its dress, or the pricking of a pin. During attacks of colic children pass their water often and in considerable quantity. Different internal causes may cause colic; such as,—1st, *Acidity*, which is known by the stools being green; in this case the following mixture is given; ℞. Inf. et aquæ Fœniculi *aa* ℥ij.; Magn. Carb. gr. xv.; Laudani Sydenhami gtt. ij.; Syrupi ℥ss.;—2dly, *flatulence*, which is known by borborygmi and wind. The following mixture is then given, with or without the addition of magnesia: ℞. Aquæ Fœniculi ℥ij.; Mucil. Acaciæ ʒij.; Laudani gtt. ij.; Syrupi Anthem ℥ss.;—3dly, *indigestion*, in which case the following mixture is prescribed: ℞. Aq. Fœniculi ℥ij.; Tr. Rhei aquosæ ʒij.; Magn. Muriatis ℈ss.; Syrupi ℥ss.

*Worms.* Dilatation of the pupil is not a pathognomonic sign of the existence of worms, for this symptom is also found in cases of infarctus of the intestines. Dr. Gölis's ordinary remedy against worms is as follows: ℞. Hydr. Subm. gr. iij.; Valerianæ pulv. ℈j.; Sacchari albi ʒj. This is divided into four or six powders, according to the age of the patient, and three are to be taken daily.

Sometimes he adds a scruple of wormwood seeds. He also prescribes the following formula: ℞. Inf. Glycyrr.; Decoct. emoll. *aa.* ʒij.; Extr. Valerianæ ℈ss.; Oxym. Scillæ ʒij. The dose is a teaspoonful.

And afterwards the following purgative: Hydr. Subm. gr. iij. Jalapæ pulv. ℈j.; Sacchari albi ℈ij. This is to be made into four or six powders, one of which is to be taken every evening.

The following anthelmintic is also employed by Dr. Gölis: ℞. Inf. Glycyrr.; Aq. Tanaceti *aa* ʒij.; Fuci Helminthocorti pulv. ℈j.; Oxym. Scillæ ʒss. A teaspoonful to be taken every hour.

In cases of ascarides clysters of milk are administered in which garlic has been boiled, or clysters of a decoction of garlic and wormwood. To draw out worms of this species, the children are placed on a chamber vessel into which warm milk has been poured.

*Infarctus of the Intestines.* In this disease Dr. Gölis gives merely resolvent medicines. ℞. Inf. Fœniculi; Aq. Fœniculi *aa* ʒij.; Potassæ Supertart. ℈ss.; Oxym. Scillæ ʒij. The dose is a teaspoonful.

Some other resolvent salt may be substituted for the cream of tartar. Externally he employs equal parts of mercurial and juniper ointment rubbed upon the abdomen; and at the same time he puts the patient on the use of acorn coffee, and tepid baths. According to Dr. Gölis there is a certain globular shape of the cheeks which is a true diagnostic sign of intestinal *infarctus* (obstruction) in children. This bulging out corresponds to the malar bones, and looks as if an almond had been placed under the skin; it is particularly obvious when the child laughs or cries. When this symptom is present, the case is hopeless.

*Icterus neonatorum* (Jaundice of new-born children.) The following prescription is used in such cases: ℞. Aq. Fœniculi; Inf. Anthem. *aa* ℥j.; Magn. Subcarb. ℈ss.; Tr. Rhei Aquosæ ʒss; Syr. Papav. ℥ss. The dose is a teaspoonful. If the bowels are confined, the following mixture may be given: Inf. Glycyrr. Aq. communis *aa* ℥j. ad ℥ij.; Extr. Taraxaci ʒj. ad ʒij.; Sodæ; Sulph ʒij.; Syr. Mannæ ℥ss. The dose is a teaspoonful.

*Prolapsus of the Rectum.* When the intestine has been reduced, Dr. Gölis orders clysters of cold lime water.

*Poisoning by Opium.* In this case he ordered a bath with vinegar of half an hour's duration, and a teaspoonful of the following mixture every quarter of an hour. ℞. Inf. Glycyrr.; Decoct. emoll. *aa* ℥j. Ammon. Succin. gtt. viij.

*Dropsies. Chronic Hydrocephalus.* It is sufficient to have seen one hydrocephalic child, to have a just notion of their physiognomy. These patients are generally known at first by a heavy and awkward manner of walking; they are apt to trip and fall, and often cross their feet. They like to have something continually in their mouth and fingers; they also put their fingers into their nose and ears; and their eyes are convulsed; this is a never-failing symptom.

If a cough accidentally occurs in the course of chronic hydrocephalus, it easily changes into fits of periodic suffocation, which has been treated of above. If the disease passes into an acute form the children soon die.

In the treatment, calomel is the chief remedy. Children under a year old take first of all an eighth of a grain, and afterwards a quarter of a grain, twice a day. At the same time their head is rubbed with equal parts of mercurial and juniper ointment, taking care to keep it warmly covered.

As far as the treatment is concerned, the most essential point in the diagnosis of chronic hydrocephalus, is to ascertain whether or not the disease is complicated with cachexy. When there is cachexy there is generally also some organic disease, as of th spleen, for example, or some *engorgement;* and it is in such a case that calomel is the sovereign remedy, particularly if the bowels are confined. Besides the use of calomel, the hypogastric and splenic regions are rubbed with the ointment mentioned above. But we must never omit to treat the cachexy at the same time. Dr. Gölis combats it with bark. One or two drachms of cinchona are to be boiled for a quarter of an hour, and eight grains of saloop then added. The boiling is then to be continued for another quarter of an hour, so that the decoction when strained may amount to four ounces, and half an ounce of syrup of poppies is then to be added: the dose is a teaspoonful every hour.

In a case of semi-paralysis, the consequence of chronic hydrocephalus, Dr. Gölis prescribed arnica in the following form: ℞. Inf. Glycyrr. et Decoct. emoll. ℥ij.; Extr. Arnicæ gr. iv.; Oxym. Scillæ ʒij.

*Partial external Hydrocephalus.* This disease, which is also called œdema of the scalp, is cured to a certainty by the occasional application of a caustic to one of the œdematous points; the best is lunar caustic. The absorbing power of the lymphatics is excited by this kind of irritation. Dr. Gölis generally moistens the swelling and then touches it in several places with the nitrate of silver; and if the result is not satisfactory at the end of a few days, he has recourse to caustic potash, of which he takes a few frustula, and applies them to various points by means of adhesive plasters. Frictions with mercurial ointment are likewise employed. Lastly it is right to keep up a uniform temperature by means of bags filled with aromatics.

If the œdema is confined to a small spot, it may be opened; but this would be dangerous if the case were complicated with internal hydrocephalus.

In a case where œdema of the scalp had followed the suppression of a porrigo, we saw Dr. Gölis administer calomel, perhaps with the intention of preventing a dropsy of the ventricles of the brain.

*Hydrorachis.* This disease is characterized by a peculiarity in the walk of the children affected by it: they totter as they go; at each step they turn their feet inwards, and always put the heel to the ground first. These peculiarities increase from day to day, and the little patients end by being unable to walk alone, and their gait always retains the characteristics which we have pointed out Hydrorachis is most usually the result of myelitis, (inflammation of the spinal marrow.) Calomel is the principal remedy, to which are added blisters to the loins.

*Ascites.* In this species of dropsy Dr. Gölis gives three doses of cream of tartar a day, together with the following potion: ℞. Inf. Glycyrr.; Aq. Fœnic. *aa* ℥ij.; Extr. amari gr. xij.; Sp. Æther. Nitr. gtt. xv.; Oxym. Simpl. ℥ss. At the same time he orders the abdomen to be rubbed with a mixture of mercurial and juniper ointment.

He also prescribes the following potion: ℞. Inf. Glycyrr.; Aq Fœnic. *aa* ℥ij.; Potassæ Subcarb. gr. ss.; Aceti Scillæ ʒiss.; Laud. Sydenh. gtt. ij. When worms are present at the same time, he prescribes: ℞. Aq. Fœniculi ℥iv.; Extr. Scillæ gr. j.; Camph. gr. iss.; Syr. Fœnic. ʒiv. And when the dropsy is complicated with scrofula, ℞. Inf. Glycyrr.; Decoct. emoll. *aa* ℥ij.; Extr. Conii gr. viij.; Potass. Acet. liquidæ ʒiss.; Oxym. Scillæ ʒiij. The dose to be a teaspoonful every hour.

*Scrofulous Diseases.* Whether the scrofulous disease be in its commencement, or already established, Dr. Gölis always gives the following powder, after having first alleviated the most urgent accessory symptoms: ℞. Test. Præp. ℥ss.; Guaiaci resinæ; Limaturæ ferri *aa* ʒss.; Sacchari albi ʒiij. The dose of this powder is a pinch (greater or smaller, according to the age of the child,) taken twice a day.

Such is the powder which Dr. Gölis prescribes at the hospital.

In private practice he substitutes his anti-hectic scrofulous powder which is made of equal parts of laurel berries, nutmeg, and hartshorn shavings. But the berries must first be deprived of their acrimony, which is done by baking them in bread. The antihectic scrofulous powder is then administered with equal parts of liquorice powder, in the dose of a large pinch three times a day, or in the following form: ℞. Pulv. Anti-hect. scrof. Gölis. ij. (vel plus); Guaiaci resinæ; Limaturæ ferri *aa* ℥ss.; Sacchari albi ℥ij. All these powders may be continued for a length of time; but whenever any inflammatory disposition exists, the resin of guaiac is omitted. In addition, the children are to have a bran bath three times a week. The diet consists of broth and milk.

*Scrofulous Exanthema.* In this case Dr. Gölis gives his ordinary powder, taking care however to substitute a scruple of antimonial æthiops (sulphuret of mercury and antimony) for the iron filings; and giving at the same time wild pansy tea for drink. If there is a syphilitic taint, instead of the iron filings, he gives the black sulphuret of mercury.

*Scrofulous Eruptions of the Head.* (Achores Scrofulosi.) No particular remedy is employed against eruptions of this kind; on the contrary, it is well if they appear, for the scrofulous disease is terminated the sooner. If an eruption on the scalp dries up quickly, the neighbouring glands are apt to swell and ultimately suppurate. There is a close connexion between the abdominal glands and the head; an eruption on the latter often disperses the *engorgement* and induration of the abdominal glands, and these glandular affections may sometimes be cured by producing an artificial eruption on the head, by means either of powdered cantharides, or by the greyish dust which is obtained by currying horses.

*Bony Tumours of a Scrofulous Nature.* Tumours of this kind are principally observed on the fingers and toes, and sometimes on the feet and arms. The tumour grows larger, becomes round and red, and ultimately opens. It is then seen that the bone is carious. Sequestra come away by degrees, and whole phalanges may be destroyed. Nevertheless, the disease gets well, but slowly, in eighteen months or two years. The limbs, though shortened, can again be employed. We must carefully abstain from stimulating treatment when we have to do with ulcers arising from scrofulous caries; local stimulants would merely aggravate the disease. The best remedies are emollient fomentations, cataplasms, and bran baths; and the medicines recommended against scrofula are to be employed internally, and also coltsfoot tea. This method often succeeds extremely well.

When it is a simple scrofulous ulcer, Dr. Gölis generally orders it to be sprinkled with equal parts of powdered rhubarb and powdered charcoal, and sometimes with the latter alone; and for lotions he uses an infusion of scordium, (Teucrium Scordium.)

If scrofulous tumours appear behind the ear, they must be opened as soon as possible, as they quickly cause caries of the mastoid process.

*Otorrhœa.* This flux almost always depends on a scrofulous taint, and must be treated accordingly. Externally nothing is to be used but a weak decoction of bran as a lotion; if caries exists, an infusion of mallow and scordium is to be employed as an injection. Styptic remedies, used with the intention of stopping the discharge, might easily produce hydrocephalus.

*Subcutaneous Lymphatic Tumours.* Tumours of this kind soon yield to the employment of the nitrate of silver, with which they are touched in the manner mentioned when treating of partial external hydrocephalus. Besides this, caustic emollient fomentations are used. If cachexia or slow fever exists, these affections are separately treated. (See *Slow Fever* afterwards.) When the tumours are opened, if they look ill they are sprinkled with a mixture of finely-powdered rhubarb and charcoal, as in the case of scrofulous ulcers.

*Sanguineous Tumours of the Head.* Dr. Gölis recommends us not to open these tumours when met with in new-born children, and asserts that children often die in consequence of such an operation. He touches them with lunar caustic, like lymphatic tumours and œdema of the scalp; and asserts that they easily yield to this remedy. He also employs the same method in cases of nævi materni, and removes them by thus exciting suppuration.

*Rickets.* This disease is sometimes ushered in by a state of weakness before there is any visible deformity of the osseous system. The children neither can nor will go upon their feet; and they cry and groan when lifted; this is the first stage of the disease. At a later period the patients begin to breathe with difficulty; and they are often seized with fits of periodic suffocation. They perspire abundantly, especially about the head: the state of rickets has now begun.

Rickety children have a peculiar manner of holding up their legs when they are lying at ease upon their back. They keep them crossed and draw them up, so as to enclose the abdomen between the thighs. Their urine has a specific odour—that of mice; their weeping and cries have something characteristic, when the disease is fairly established; and an experienced ear may always guess the disease by this sign alone. They are seldom thirsty, even when suffering from an inflammatory fever, with or without local affection. Their head is frequently very large, besides the deformity which often occurs in this part, and they have generally more capacity than other children. It is very seldom, indeed, that they are affected with hydrocephalus.

Some rickety children, in spite of their disease, present the aspect of health; this is *rachitis florida.* Such children are usually of a scrofulous habit, and in them the malady probably originates, from a real want of osseous matter. The treatment is the same in this case; but the prognosis is more favourable, and the little patients are sometimes quickly cured in spring or summer. Before beginning the treatment, all the accessory symptoms, such as cough, diarrhœa, &c. must, of course, be removed; and Dr. Gölis then generally prescribes the following powder with the best results. ℞. Testarum præpar. ℥ss.; Ferri limaturæ, ʒss.; Sacchari albi, ʒij.

The dose is a pinch morning and evening. A bath of hay flowers is to be taken at the same time, three times a week. The diet consists of acorn coffee, with milk once or twice a day, broth, and meat; farinaceous food is forbidden. He does not allow them to sit, or to be much carried in arms; the horizontal position is the best, but they must not lie on feather beds.

It is an error, according to Dr. Gölis, to suppose that the oxides of iron are borne more easily than the filings. He has never succeeded with madder in the cure of rickets, even when he has used it with great perseverance. The most troublesome complication of this disease is hooping-cough.

*Chronic Tension of the Skin.* (Cutis tensa chronica.) This disease, though not very rare, is not at all known; it is characterized by a peculiar tension, of a shining red, of the skin of the face, particularly around the mouth; or of the hollow of the hands, the soles of the feet, and the upper and interior part of the thighs. By degrees the stretched parts become harder and wrinkled: the lips become covered with crusts, which sometimes extend to the cheeks, and under these crusts there is an acrid humour, which corrodes the flesh in patches. Ulcerations also form on the thighs, around the genitals, on the soles of the feet, and on the hands.

This disease usually depends on a syphilitic taint, and the treatment is perfectly in accordance with this theory: calomel is the only remedy, the specific. Dr. Gölis prescribes it in the dose of a quarter or a third of a grain, according to the child's age, three times a day. He gives a decoction of wild pansy in milk for drink, and orders a bran bath occasionally. The disease, however, is rarely, if ever, cured without leaving traces behind, especially about the mouth. Caries, or some other disease of the osseous system, is a frequent consequence. In less serious cases the disease degenerates into a crusta lactea.

*Blue Fever.* (Febris cærulea.) The disease, which Dr. Gölis designates by this name, is an affection *sui generis*, that has not yet been described, and must not be confounded with the *morbus cæruleus*, which is caused by a disease of the heart. It occurs only in children between four and twelve months old, and usually among those of the poor, who are fed on coarse farinaceous food, and live in damp unwholesome dwellings. The disease occurs in paroxysms only; the children turn blue suddenly, their breathing is painful, their pulse small, hard, and spasmodic. The paroxysm lasts for some time, disappears, and returns again; but the intervals are shorter from day to day, and at last the paroxysms become continuous. The skin is often covered with clammy perspiration, and death takes place suddenly. On inspection, the vessels are found gorged with blood. With the exception of the fever, which accompanies the paroxysms, the disease has all the characters of a neurosis, and the medicines from which Dr. Gölis has derived most advantage, are the succinate of ammonia and other antispasmodics, combined with mucilaginous remedies. Here is his formula: ℞. Aq. Anthemidis, ℥ij.; Ammon. succin. liquid. gtt., vj.; Laud. Sydenhami gtt. j.; Tr. Castorei gtt. vj.; Mucil. Acaciæ; Syrupi Papav. *aa* ℥ss. The dose is a teaspoonful every hour. In addition to the internal remedies, he orders tepid alkaline baths, half a gallon of ley being added to a bath.

When the spasm is relieved, it is proper to give calomel as a purgative, or a mixture of rhubarb and magnesia.

Children moreover are subject to a particular chronic sweating, which is not the disease called by the French *la suette*, because it is unaccompanied by fever. Here the skin becomes blueish, and, as it were, transparent. This may be combated, according to Dr. Gölis, by the administration of a light infusion of bark and milk, and frictions, with almond oil, repeated several times a day.

*Slow Fever.* Slow or hectic fever is, as every one knows, the result of very different diseases; emaciation of the neck is one of the first symptoms. Dr. Gölis here gives acorn coffee, or a decoction made by boiling one or two drachms of cinchona for a few minutes, and then adding eight grains of saloop. This is again boiled for a few minutes, so that the strained liquor may amount to four ounces, and then half an ounce of syrup of poppies is added. The dose is a teaspoonful every hour. According to circumstances he orders the following ointment: ℞. Ung. Althææ, ℥ss.; Ung. Hydr. ʒij.; M.

A piece, the size of a haricot bean, is to be rubbed upon the abdomen morning and evening. In the morning, the children have acorn coffee; at noon and in the evening, a panada, with the yolk of an egg or ground rice. When the hectic fever is in an advanced stage, and is accompanied by diarrhœa, Dr. Gölis prescribes as follows: ℞. Decoct. Althææ rad.; Inf. Glycyrr. *aa* ℥ij.; Laud. Sydenhami gtt. ij.; Ammon. Succin. liquid. gtt. xv. Sometimes, also, an equal quantity of rue water is substituted for the decoction of mallow.

On the following formula is prescribed: ℞. Aq. Tiliæ flor., ℥ij.; Mucil. Acaciæ, ʒij.; Ammon. Succin. liquid, gtt. xv.; Syr. Papav., ℥ss.; M. The dose to be a teaspoonful every hour.

When scrofulous children are attacked by hectic fever, the disease terminates in hydrocephalus six times out of seven.

*Intermittent Fever.* Dr. Gölis often treats this disease with perfect success, by giving fifteen grains of powdered oyster shells alone, three times a day. He also prescribes: ℞. Inf. Glycyrr; Decoct. Althææ *aa* ℥j. ad ℥ij.; Ammon. Muriatis, ℈ss.; Extr. Taraxaci, ʒj.; M. Sum. coch. j. max. 2 q. horâ.

If the case is complicated with visceral *engorgemens*, he uses frictions with equal parts of Ung. nervinum, and mercurial ointment.

NEUROSES. *Convulsions of new-born Children.* These convulsions are usually caused by cerebral irritation; and Dr. Gölis always opposes the use of stimulating antispasmodics, which, he says, only aggravate the disease. Antiphlogistics alone are suitable, namely, calomel in small doses, baths, and emollient clysters.

*Catalepsy.* The author of this article saw but one case of catalepsy while attending Dr. Gölis's practice, and this case was complicated with worms. After a mild aperient, calomel was administered in combination with valerian.

*Epilepsy.* In a case of epilepsy, Dr. Gölis prescribed as follows: ℞. Test. præp. pulv., ℥ss.; Valer. rad. pulv.; Ferri limaturæ *aa* ʒss.; Sacchari albi, ʒiij. The dose to be two or three pinches a day.

*Trismus.* Dr. Gölis has never been able to save a new-born infant attacked with this disease.

DISEASES OF THE SKIN. *Scarlatina.* Dr. Gölis's treatment changes according to the character of the fever. When the eruption comes out but imperfectly, the body is ordered to be washed with tepid water. Dr. Gölis extols this proceeding extremely, and never substitutes diaphoretics for it in the beginning, as they might prove injurious.

*Measles.* When scrofulous children are attacked with this disease, it is apt to give rise to hectic fever. Measles, like scarlatina, must be treated according to the character of the fever and other circumstances, sometimes by antiphlogistics, and sometimes by diaphoretics. The following is Dr. Gölis's diaphoretic prescription in this case: ℞. Aq. Tiliæ flor., ℥iij.; Liq. Ammon. Acet., ʒj.; Syr. Althææ, ℥ss.

*Tinea Capitis.* (Porrigo). Tinea, like the crusta lactea and herpes, is frequently of a scrofulous origin; for as the scrofulous virus produces the crusta lactea on the face, so it causes tinea on the head, and herpes on the body. It is worthy of remark, that if a well-dried porriginous crust is powdered, and another person is rubbed with this powder on any part of his body, the spot thus rubbed becomes covered with an eruption.

The internal treatment of porrigo is that which is suited to the scrofulous affection. A decoction of wild pansy in milk is given for drink, and an ointment is rubbed on the dry crusts, consisting of fifteen grains of red precipitate to half an ounce of fresh butter. Emollient fomentations are used to soften the crusts, and make them fall off.

In a case of affection of the chest, arising from a suppressed porrigo, Dr. Gölis prescribed as follows: ℞. Test. Præp., ℥ss.; Guaiaci resin., ʒss.; Hydr. & Antim. Sulphureti, ℈j.; Sacchari albi, ʒiij. A pinch to be taken morning and evening.

*Crusta Lactea.* (Porrigo larvata.) In this eruption, Dr. Gölis always employs an anti-scrofulous treatment. In this, as in all other eruptions of a scrofulous nature, coltsfoot is to be preferred to the wild pansy. The crusta lactea sometimes changes to a serpiginous crust; but in such cases a syphilitic taint is always to be suspected.

*Chronic Pemphigus.* An anti-scrofulous treatment is proper in this disease likewise.

*Itch.* There is a species of itch, or rather of psoric disease, which is not contagious; it is often observed after vaccination. Scrofulous itch too is not infectious.

The antipsoric treatment of Dr. Gölis is as follows: internally he prescribes—℞. Magnesiæ Muriatis; Sacchari albi, *aa* ʒij.; Sulph. sublim., ʒj. Half a teaspoonful, or more, to be taken three times a day.

Coltsfoot tea is to be given as drink, and a mixture of sulphur ointment and soap is to be rubbed on externally. These frictions are to be performed twice a day, on the parts where there are no psoric pustules.

*Intertrigo.* (Chafing). The following is Dr. Gölis's method of treating this erythema, which occurs in children between the thighs and around the genitals. He foments the affected parts with the following mixture: ℞. Aq. Calcis, lbss.; Plumbi Acet. gr. xv. At the same time, he endeavours to excite an eruption on the head by means of Garou's ointment. Internally, he administers calomel in the dose of a quarter or half of a grain, and gives wild pansy tea.

If the intertrigo is of a syphilitic nature, the fomentations are made with the yellow wash consisting of a grain of corrosive sublimate to four ounces of lime water.

*Aphthæ.* In this disease he prescribes ℞. Mellis Rosæ ℥j.; Boracis, gr. xv. M. This is to be applied to the affected parts four times a day.

The following formula is also used: ℞. Mellis Rosæ ℥j.; Syr. Mori ℥ss.; Boracis ʒss. When there is much inflammation of the mouth the borax is too irritating, and the honey alone should be used. We must never lose sight of the fever by which aphthæ may be accompanied. The salivation induced by the disease often causes dyspepsia, which is remedied by extract of dandelion, dissolved in an aromatic water.

### ULCERATION OF THE NECK OF THE UTERUS.

DR. MAGISTEL has given eight cases of this disease in the *Gazette Médicale*. In the first one, the patient was a lady aged 38, and the disease was caused by wearing a pessary. A cure was effected, in two months, by removing the instrument, employing emollient injections, and by one application of a solution of the nitrate of mercury.

CASE II. A lady who had long suffered from leucorrhœa, and had formerly been affected with venereal sores, put herself under the care of our author. By means of the speculum two irregular ulcers were detected on the cervix uteri. Sarsaparilla and corrosive sublimate were prescribed, and the ulcers touched with creosote. As the creosote seemed to do no good, the nitrate of mercury was substituted, and the tubercles around the sores were touched with nitrate of silver. Injections were thrown up twice a day, and the vagina was plugged to keep its parietes asunder. The patient was completely cured in ten weeks, during which she had taken twelve grains of the corrosive sublimate. The gums were scarcely swelled. The cicatrices of the ulcers were of a blueish colour.

Dr. Magistel observes, that since he treated this patient he has twice employed creosote without success: some benefit was apparently produced for the first few days, but the ulcer soon reassumed its habitual state.

Case iii. A lady, aged 24, who had been married but six months, complained of heat, pruritus vulvæ, and a mucous leucorrhœa. Examination disclosed an ulcer at the neck of the uterus, as well as some excoriations of the vulva. A cure was easily effected by twelve leeches applied to the anus, injections of decoction of mallow, topical baths, acidulated drink, and the insertion of plugs in the vagina, which were renewed twice a day. The discharge had nearly ceased at the end of six days. The ulcer was then touched with the acid nitrate of mercury, which healed it in ten days more. Decoction of hemlock was now injected, and ten grains of black oxide of iron taken daily; and an infusion of hops for diet drink. The first time that the catamenia returned, they were followed by a whitish discharge which lasted two days; the leucorrhœa has not appeared again, but our author has warned his patient that the smallest error in regimen would cause a relapse. Dr. Magistel thinks that this was a case of primary leucorrhœa, produced by amorous excess, and the neglect of cleanliness.

Case iv. A lady, aged 42, who had borne seven children, and had long been subject to leucorrhœa, was attacked with violent menorrhagia on the 21st of May, 1834. A bleeding, a mustard poultice to the back, the infusion of ratanhy, and tannin pills, afforded temporary relief; but the hemorrhage recommenced the following day, on the patient's getting out of bed. Cold aluminous injections, and the application of ice to the hypogastrium, stopped it for a time, but during the night it returned with greater violence than ever. During our author's absence, one of his friends prescribed the ergot of rye, in the dose of eight grains every two hours. The hemorrhage, however, still continued, and about eight in the morning, Dr. Magistel found the patient in a state of extreme prostration, with a thready pulse, and a yellow tint on her countenance. The vagina was now plugged. Three days afterwards the patient had a shivering fit: the plug was removed, and some fetid matter issued forth, whose partial re-absorption may have been the cause of the shivering. On the 25th of May, a large ulcer was discovered on the cervix uteri, and on the 1st of June, it was cauterized for the first time with the nitrate of mercury. Injections of a decoction of mallow and hemlock were employed immediately after the application of the caustic, and the plug was employed twice a day until the 10th of July. On the 2d of August the mucous membrane of the vagina was found to be healthy, and the ulcer entirely healed. The catamenia also re-appeared, but the use of the speculum always detected a white and viscous discharge at the orifice of the uterus.

This patient was soon cured by injections of conium morning and evening; pills composed of one grain of conium, and one grain of extract of aconite, (of which she sometimes took as many as six a day;) decoction of dulcamara; cupping on the sacrum and loins; tepid baths; Seidlitz water in small doses; enemata; the employment of the plug and of caustic; repose and vegetable diet.

Case v. Madame R. æt. 46, who had long suffered from leucorrhœa, and a reddish discharge, was attacked in the beginning of March 1834, with violent uterine hemorrhage. When our author was called in, the patient had already been bled, and cold injections, the application of ice to the hypogastrium, five grains of tannin taken in pills, sinapisms between the shoulders, and the infusion of ratanhy, had been used in vain. Dr. Magistel ordered the patient to lie down with the pelvis higher than the trunk, and prescribed 60 grains of ergot of rye; ten grains to be taken every two hours in sugar and water. The hemorrhage ceased after the fourth dose. An ulcerated scirrhus of the cervix uteri was afterwards discovered, which was removed by operation, and the patient recovered perfectly.

Our author remarks, that in the former case the ergot of rye failed in arresting the hemorrhage, but succeeded in this one, which he explains by the hemorrhage in Case iv., proceeding from vessels opening into an ulcer; while in the one just narrated, it arose from the internal parietes of the uterus.

Case vi. A lady, aged 60, who had suffered from syphilis in her youth, sunk under cancer of the uterus, unattended with hemorrhage, which had lasted fifteen years. Our author narrates this as a remarkable case of cancerous diathesis; there was a cancerous pimple on the lower lip, a mass of cancer in the rectum, and the mucous membrane of the stomach crackled under the knife. He attributes the origin of the disease to the abuse of mercury.

The 7th case is that of a lady of rank, in whom a temporary cure of cancer of the uterus was effected by antiphlogistic treatment. The 8th is one of a druggist's wife suffering from the same disease, and concealing it from modesty. She died of hemorrhage.

The following are the author's general conclusions.

1. When there is the slightest symptom of uterine disease, the uterus must be examined with the speculum.

2. When ergot of rye has failed in arresting hemorrhage from the uterus, we ought to have recourse to plugging, without going through the general remedies employed in such cases.

3. The slightest ulceration of the uterus must be treated by caustics.

4. The best caustic in these cases is the supernitrate of mercury.

5. In chronic catarrh of the vagina, the employment of a plug of charpie permanently remaining in the vagina may be of great advantage, by keeping the parietes asunder, and absorbing the secretion, either from the mucous membrane, or from ulcers, if there are any.

6. Women may sometimes live a long time with cancer of the uterus, if there is no hemorrhage.

### TIC DOULOUREUX.

There are two papers on this subject in the 28th Number of the *American Journal of the Medical Sciences.* The first is by Dr. Chapman, who enters at some length into the history and varieties of the disease, and discusses the numerous remedies which have been proposed for its cure. Among these is galvanism applied in the method directed by Mr. Mansford, which the author affirms to have been lately employed with success by his friend, Dr. Harris, in three cases of the disease. As this singular method is quite unknown in this country, we shall give it at length.

"It was said that, in order to fulfil the indication stated at the commencement of this section, it was desirable to establish a negative point as near the brain as possible, and a positive one in some distant part of the body. Accordingly, a portion of the cuticle, of the size of a sixpence, being removed by means of a small blister on the back of the neck, as close to the root of the hair as possible, and a similar portion in the hollow beneath, and on the inside of the knee, as the most convenient place: to the wound in the neck a plate of silver, varying, according to the age of the patient, from the size of a sixpence to that of a half-crown, was applied, having affixed to its back part a handle or shank, and to its lower edge, and parallel with the shank, a small staple, to which the conducting wire was fastened. This wire descended the back till it reached a belt of chamois leather, buttoned round the waist; it then followed the course of the belt, to which it was attached, till it arrived opposite the groin on the side it was wished to be used; it then passed down the inside of the thigh, and was fastened to the zinc plate in the same manner as to the silver one. The apparatus so contrived was thus applied: a small bit of sponge moistened in water, and corresponding in size to the aperture in the neck, was first placed directly upon it; over this a larger piece of sponge, of the same size as the metallic plate, also wetted, was laid; and next to this the plate itself, which was secured in its situation by a strip of adhesive plaster passed through the shank on its back, another above, and another below it. If these be properly placed, and the wire which passes down the back be allowed sufficient room, that it may not drag, the plate will not be moved from its position by any ordinary motion of the body. The zinc plate was fastened in the same manner; but, in place of the second layer of sponge, a bit of muscle, answering in size to the zinc plate, was interposed; that is, a small bit of moistened sponge being first fitted to the aperture below the knee, the piece of muscle, also wetted, then followed, and on this the plate of zinc. The apparatus thus arranged will continue in gentle and uninterrupted action from twelve to twenty-four hours, according to circumstances. This last is the longest period that it can be allowed to go unremoved: the sores require cleaning and dressing, and the surface of the zinc becomes covered with a thick oxide, which must be removed to restore its freedom of action: this may be done by scraping or polishing; but it will be better if removed twice a day, both for the greater security of a permanent action, and for the additional comfort of the patient." (*Mansford on Epilepsy.*)

Dr. Chapman observes, that parchment or buckskin may be advantageously substituted for the piece of muscle.

The author gives three cases in which the neuralgia depended on irritation of the teeth, and was cured by extraction of the peccant tooth or stump. Dr. Chapman then gives six cases, depending on more or less obvious irritation of the spine. These were cured or relieved by leeches, moxa issues, and blisters applied to the morbid part.

Three other cases are attributed by the author to ganglionic irritation. The first was an elderly gentleman, who had lived in a "miasmatic district of country," but had escaped the ague. In place of this, however, he was afflicted with cramps of the stomach, succeeded by darting, poignant pains through the hypochondria, in the region of the kidneys, and of the bladder.

"The case had perplexed various physicians exceedingly, having been considered as gout, chronic hepatitis, nephritis calculosa, stricture of the urethra, &c. Being satisfied, on a careful perquisition, that these conjectures were incorrect, I inclined to believe that the case was of a neuralgic nature, originating in miasmatic influence; and the more so from having seen, during the prevalence of epidemic intermittent fever among us, which abounded in anomalies of all sorts, not a few similar instances. No tenderness, however, could I detect in any part of the spine. nor were there any symptoms referrible to that source of irritation.

"Correcting the disorder of the chylopoïetic viscera, which was conspicuous, by an alterative course of the bluepill, and the occasional interposition of a purge, I then administered largely the sulphate of quinine, alone, and with the piperine. These remedies were useful, though not decisive, and I next resorted to the sulphate of copper, which completely put an end to every affection."

In the second case, the patient complained of cramps of the stomach and bowels, with occasional darting pains through the abdomen. He lost about sixty ounces of blood by venesection and leeching, with evident aggravation of the symptoms. The subnitrate of bismuth, combined with opium, was then given with some advantage, and the cure was completed by the carbonate of iron.

In the last case the patient was gouty, and the disease yielded to colchicum.

The other paper on the same subject is by Dr. Harris, who narrates eight cases of neuralgia treated by Mansford's galvanic apparatus. The results we will give in his own words.

"Of the eight cases which have been treated with the galvanic agent, five have been entirely relieved. In every case in which the disease was located in the cerebral nerves, this simple and apparently feeble galvanic apparatus has accomplished cures. On the contrary, where this disease was diffused throughout the body, or located in the spinal nerves, it has failed."*

* This Number of the American Journal contains a case of paraplegia, and a case of general paralysis, occurring in the Baltimore Almshouse Infirmary, and successfully treated by Dr. J. H. Miller, with the same remedy.—Ed. *Med. Quart. Rev.*

### CASE OF ATRESIA OF THE VAGINA, PRODUCED BY IMPROPER TREATMENT.

By Thomas Jefferson White, m.d.

A case of this kind occurred to me last summer, during the prevalence of epidemic cholera, from the malpractice of an ignorant empiric of the Thomsonian fraternity.

The subject of it was a negro girl, robust and healthy, æt. thirty, who had been attacked with spasmodic cholera, but had recovered entirely, the system merely laboring under the slight febrile excitement which usually succeeds those cases. I was sent for repeatedly, to obviate that excitement, but an extensive practice prevented me from visiting the patient. I however requested one of my students to call, and administer a refrigerant; in the mean time Mr. B. of steam memory, had prevailed on the owner to permit him to take her through a course of No. 6, &c., telling him that the spasms would recur, unless that plan was adopted. The owner, alarmed at the character of the epidemic, and the number of cases in his family, readily consented. Eleven emetics of lobelia were administered successively, and twelve injections of the infusion of red pepper, some or all of which were thrown up the vagina instead of the rectum. The consequence can be readily imagined. There were great prostration from the lobelia, and an active and extensive inflammation of the mucous coat of the vagina, with an entire sphacelus of that membrane, from the distressing effects of the red pepper, together with a total adhesion of its parietes, with the exception of a very small space at the posterior portion of it, which could be barely reached by the point of the hidden finger.

To remedy this, a perpendicular incision, an inch and a half in width, was made, making the hidden finger of my left hand a director, and cut through the occluded portion of the vagina gradually and cautiously; taking care to avoid the bladder on the one hand, and the rectum on the other, until the knife reached the space between the occluded portion of the vagina and mouth of the uterus, the incision being thus upwards of five inches in length. A large quantity of the catamenial fluid was discharged, to the great relief of the patient, who had suffered for several days the most excruciating pain I had ever witnessed.

A thick bougie was then introduced, previously smeared with ceratum simplex, and this was retained until the parts healed completely, except when occasionally removed for purposes of cleanliness. No inconvenience has resulted from the operation, and she has even menstruated freely, and is in perfect health.

I conceive it due to myself, my profession, my country, and humanity, to publish the history of the above case, imperfect as it is. It ought to stigmatize with infamy the ignorant pretender, who has thus abused the principles of the most godlike and useful of sciences.—*Baltimore Med. and Surg. Journal.*

### USE OF PURGATIVES.

There is a curious paper on this subject in the *Archives Générales* for September. The author, Dr. Simon, after having theoretically considered the effect of purgatives in a previous Number, gives in this one an account of their therapeutic effects, when administered to a number of M. Andral's patients at the *Pitié.*

Case I. A carter, aged sixty-one, was suffering from bronchitis; his pulse was 116, and his respirations twenty-six in a minute. He was bled, took the gum-drink, with infusion of violets, and was put on low diet. These remedies produced no effect, and the patient remained in the same state for five or six days, when a violent fit of dyspnœa supervened. The day after this attack, the pulse was 108, the respirations twenty-four, and there was a decided mucous *râle*, both anteriorly and posteriorly. A jug of barley-water was now prescribed, with an ounce of sulphate of soda, and half a grain of tartar emetic. He was also allowed soup and broth. The aperient produced ten stools, and the pulse immediately fell to eighty-four, and the respirations to sixteen; the *râles* too were confined to the posterior part of the chest. The patient now quitted the hospital.

Case II. A turner, aged fifty-three, was admitted, suffering from hypertrophy and dilatation of the heart, with chronic bronchitis, slight ascites, considerable anasarca of the legs, and slight œdema of the arms. The pulse was very rapid, and so irregular as scarcely to admit of being counted. Respirations, twenty-eight. The treatment began by bleeding; there was no buff on the blood.

The pulse remained as before, and so did the œdema. Twelve grains of calomel were now prescribed, which produced four very liquid stools; the pulse, which could now be counted, fell to eighty-four, and the respirations to twenty-two. After the interval of a day, a jug of barley-water was prescribed, with an ounce of sulphate of soda, and half a grain of tartar emetic; this produced seven copious evacuations. The pulse remained at eighty-four, the respirations fell to twenty, and the anasarca was much diminished. On the following day, the pulse was seventy-six, the breathing much easier, and the cough much less.

The third case was one of pulmonary emphysema and chronic bronchitis, and the purgative consisted of two drops of croton oil, which produced great relief.

The fourth case was one of chronic bronchitis, with pulmonary emphysema, and slight hypertrophy of the heart. Two drops of croton oil were administered, which produced eighteen stools. The relief was great and immediate; the pulse falling from one hundred to sixty-eight, and the respirations from thirty-six to twenty.

The fifth and sixth were cases of pleurodyne, treated with leeches and purgatives: in the first of the two, the purgative was of no benefit; but the plain fact is, that the pulse was only sixty-eight, and a blister was obviously indicated from the commencement.

The seventh case is termed by our author chronic laryngitis. The pulse was eighty, and the respirations twenty. Bleeding and leeches were tried in vain, and friction with croton oil was of temporary advantage only. The patient was cured partly by a spontaneous diarrhœa, and partly by two doses of croton oil.

This paper is interesting, both as showing how necessary a French physician thinks it to apologise for a method of treatment familiar to every British practitioner; and also as demonstrating the utility of that method in the clearest manner; for the purgatives and the venesection being used on separate days, their respective merits cannot be confounded.

### CASES OF DIABETES AND CALCULUS RENALIS.

Dr. Lefevre, physician to the British embassy at St. Petersburg, has published, in the *Medical Gazette*, the particulars of a case of diabetes mellitus, successfully treated by him two years since. The patient was a woman, about fifty years of age, several of whose family had died of dropsy. She passed about fifteen pints of urine daily, was tormented by continual thirst, and the skin was dry and rough. The treatment consisted of two bleedings, animal diet, Dover's powder, and the warm bath. The bowels were kept open by calomel and colocynth, and she took equal parts of milk and limewater for common drink. These remedies mitigated the diabetes, but were unable to restore the perspiration. The cure was perfected by two vapour-baths, which caused the most profuse sweating, and the patient has suffered no relapse. Dr. Lefevre was at that time unacquainted with the vapour-bath, except as a medical agent, but a residence of several years in St. Petersburg has familiarised him with it, as a means of cleanliness used by all classes of people.

"The vapour-bath is a *sine qua non* of a Russian boor's existence. The soldiers and sailors use it twice, and the peasants at least once, a week. Baths abound in the capital; and in every village where there are twenty houses there is one devoted to the purposes of a bath. People of all ages use them, and the heat, generated in various ways, soon throws the person into a profuse perspiration. Sometimes dry heat is employed, and the individual stands in the bath as in an oven. Vapour is produced by dashing cold water upon hot stones. The bather generally lies upon a plank in the hottest part of the bath, and is flagellated with soft rods, or he is scraped down with a kind of hoop, or rubbed with shavings or hair brushes.

"Whichever of these processes he may undergo, and he has his choice, as also of his grooms, the effect produced is a profuse perspiration from every pore. When this has continued a certain time, warm water is dashed all over him, and then water a little cooler, and finally, water at a very low temperature. This is succeeded by dry rubbing, which produces a genial glow; and as the operation is generally performed of an evening, he retires from the bath to the bed or couch, and perspires moderately for the remainder of the day."

It seemed probable to Dr. Lefevre that the frequent use of these baths, by forcing the skin to perform its own office, would prevent the necessity of its being vicariously executed by the kidneys; and, on inquiry, he found that diabetes was a disease hardly known in Russia. Sir James Wylie, who has inspected upwards of two millions of soldiers, had never seen a case; nor had Dr. Freennius, a physician of great eminence. No instance of this disease is recorded in the books of the civil or military hospitals for the last twenty years.

Dr. Lefevre concludes by observing, that people bathe less in England than any where else; an assertion which is unfortunately but too true. Far from the poor being able to afford a bath, even persons of the middle class often allege the expense in defence of their unwashed state. So that the affair stands thus: myriads are unwashed because the price of cleanliness is too high, and the price is not lowered, because the dealers in cleanliness do not believe in the existence of a large class having a real taste for bathing. The remedy for this state of things would consist in some benevolent and opulent person starting a few bathing-houses, careless whether he lost or not by them. The price of a bath might be a shilling, or less; and we think that the founder of these establishments would be a gainer.

The second case is one of a renal calculus. The patient first consulted Dr. Lefevre in August, 1833, complaining of nothing but the colour of his urine, which was of the deep tint of porter; but this symptom was followed by a succession of nephritic attacks during the space of eight or nine months. The pains in the region of the kidney now vanished, and the sole uneasiness was in the bladder. The patient refused to be sounded, and set out upon a journey; he suffered a good deal from the jolting of the carriage, and a suppression of urine took place, while it could be felt externally that there was a foreign body in the urethra. We will conclude the case in the words of the author:

"The medical man who came to his assistance extracted a rough-shaped calculus, which was broken in the operation, and a considerable portion of it was lost. The extraction of it created great local pain, but this was not succeeded by any constitutional irritation, and the patient soon felt relieved from all his complaints. The portion of the calculus which he gave me for analysis weighed six grains. It was analysed by a good chemist, and was found to be oxalate of lime pure.

"It was ten months from the first uneasy symptoms which the patient experienced until the calculus passed into the bladder. True to the character of the mulberry calculus, there was never any deposit in the urine."

### DIAGNOSIS OF OVARIAN DROPSY FROM ASCITES.

The diagnosis from ascites need not be mistaken. When the ovarian tumor is within a moderate size, its circumscription is conclusive. Its commencement *on one side* has been unnecessarily insisted upon since, as its attachment is generally long and free; it soon passes towards the mesial line, where most latitude is afforded for its expansion.

When the tumor fills the whole abdomen, the diagnosis is more difficult: here, however, percussion affords excellent, and, with ordinary care, conclusive signs. In ascites, the intestines float upon the fluid, and the resonance on percussion is hollow in the most elevated situations; and invariably so in the umbilical and epigastric regions. (Piorry's ivory Plessimeter is here preferable to the hand.) On the contrary, the ovarian tumor being developed *in front* of the intestines, which it forces back, the most prominent part of the tumor is always dull on percussion. Further, to a practised ear, the dulness on percussion of an encysted dropsy is much greater than that of ascites; since, in ascites, the layer of fluid before the intestines is never so thick as to prevent a certain degree of resonance from being elicited by firm and smart percussion. Again, fluctuation is more distinct in ascites than in ovarian dropsy, unless much tympanitic tension coexist with the ascites,—a very common case: but here, fortunately, the high degree of resonance affords an unequivocal diagnostic sign. In ovarian dropsy, the neck of the uterus is usually drawn up out of reach. The general symptoms also are different, ascites being almost always connected with some old organic disease of the liver, heart, or kidneys, and attended with infiltration in other parts; while ovarian dropsy may exist independent of them, and may even be compatible with a perfect state of the general health.—*Hope's Morbid Anatomy.*

### TRACHEOTOMY.

Dr. Trousseau has performed the operation of tracheotomy thirty times, and gives the results in the following table.

| Year. | Patient's Age. | Result. |
|---|---|---|
| 1826 | 7 | Died in fourteen hours. |
| 1828 | 26 | Died in sixteen hours. |
| 1831 | 5 | Cured. |
| 1832 | 11 | Died in eleven hours. |
| — | 2½ | Died in three hours. |
| — | 5 | Died in seven hours. |
| — | 2½ | Died in fifteen hours. |
| 1833 | 3 | Died in twenty-two hours. |
| — | 4½ | Died in four days. |
| — | 6 | Died in sixteen hours. |
| 1834 | 3 | Cured. |
| — | 16 months. | Died before the operation. |
| — | 3½ years. | Died in twenty-six hours. |
| — | 4 | Cured. |
| — | 5 | Died before the operation. |
| — | 3 | Cured. |
| — | 21 | Died in three hours. |
| — | 13 months | Cured. |
| — | 4 years | Died in thirty hours. |
| — | 5 | Died before the operation. |
| — | 5½ | Died in six hours. |
| — | 4½ | Cured. |
| — | 3 | Died in thirty-six hours. |
| — | 5½ | Cured. |
| — | 1 | Died in twenty-six hours. |
| — | 2½ | Died in five days. |
| — | 3 | Died in three days. |
| — | 3 | Cured. |
| — | 5 | Died in thirty-six hours. |

This gives a total of thirty cases, among which are eight cures, and twenty-two deaths. M. Trousseau thinks we ought to subtract from the total number, not only the three cases where the patients were really dead before the operation, but three others, where the patients were going on well, but lost their lives from the awkwardness of the nurses, who were unable to replace the canula, which had been expelled by violent coughing. The number of cures is therefore eight out of twenty-four cases; by no means an unfavourable result, as it shows the possibility of saving one out of three patients where, without the operation, not one would survive.—*Journal des Connaissances Médico-Chirurgicales.*

### ISCHURIA RENALIS.

A remarkable case of this disease is narrated by Dr. de Leon, in the *Baltimore Medical Journal.* The patient was a lady, about fifty years of age, and recovered at the beginning of the fifth week apparently by the *vis medicatrix naturæ,* after a variety of treatment had been tried without benefit. Dr. de Leon says "it may be expected of me to detail the practice pursued in the case. To sum it up in a few words, I would remark, that every remedy suggested by writers on the disease had a full trial before they were laid aside. I am not therefore prepared to admit the value of one over another. Cupping over the spine, repeatedly instituted, and followed up by the most powerful rubefacients, by friction, claimed my greatest regard."

This is the second case of this disease which the author has seen. In the other instance, the patient, a gentleman aged about thirty-eight, died at the end of the third week. The bladder was much contracted, and the right kidney was considerably enlarged, and bore marks of recent inflammation.

### BELLINGERI ON NEURALGIA.

In the *Annali Universali* for April there is a memoir by C. F. Bellingeri, containing the results of his experience on this subject, during fourteen years' practice at Turin. In the course of this period he has treated 5,612 patients, of whom forty were affected with neuralgia of the different branches of the fifth or seventh pairs.

*Symptomatology.* Bellingeri has little to add to the description given by authors. Once he saw, in a case of suborbital cephalalgia of the left side, the hair become more stiff, thick, and grew more rapidly at the anterior part of the affected side than on the other. It was cured by section and cauterization of the nerve, and the hair took on its natural condition. In another case the hair fell off.

*Form and Progress.* In general, neuralgia has an intermittent but irregular progress. Sometimes the attacks are periodical; frequently neuralgia treated properly has been brought to a double tertian or quotidian type, and is easily cured with quinine. Periodical attacks, whether double tertian or quotidian, always come on before mid-day. The attacks are never periodical at first.

*Seat.* The most common seat of neuralgia is the 5th pair. Twice only it was observed in the seventh pair; in one of these instances the disease depended on a traumatic cause, and in the other it was owing to the enlargement of scrofulous glands in the vicinity of the nerves of the branches of the fifth pair: the most frequently morbid are the suborbital and frontal, the former almost invariably on the right side, the second commonly on the left. Frontal neuralgia, or that of the different branches of the ophthalmic nerve, most frequently puts on a periodic form, which is never the case with the suborbital.

*Influence of Age.* All the affected had passed forty years, with the exception of two brothers, one twenty-five and the other thirty years, whose mother had been subject for years to obstinate headachs, which were often repeated in the course of the year.

*Sex and Temperament.* Sex does not appear to influence development of neuralgia; but unmarried persons are more subject to it. The same obtains in those of sanguine, or sanguine-nervous temperaments, of athletic form, or of rigid, hard, and sensitive fibre.

*Periods of the Year.* Neuralgia almost always commences for the first time in spring or autumn, but especially in spring. It must be remarked that in Piedmont the spring is more liable to sudden atmospheric vicissitudes than the autumn. Neuralgiæ of any standing are also more intense at these seasons.

*Duration.* Old intractable neuralgia ceases spontaneously, or is considerably diminished after a period of ten or twelve years. In one case only a suborbital neuralgia of the left side carried the patient to his grave, after eight years of suffering; it was a sexagenarian priest, of sanguino-nervous temperament, and rigid, hard fibre. The disease was in the first instance cured; but he having gone into another country, the neuralgia reappeared, and resisted every remedy.

*Relapses.* In two subjects that were cured of this disease, the affection reappeared in one of them after two years; in the other, after ten years. They were a second time cured. In both the relapses was to the nerve primarily affected, but of the opposite side. Bellingeri has cured thirty-six acute neuralgiæ, which he has treated from their commencement. In the majority of these the disease only relapsed after several years; in others, the cure appeared consolidated. The other cases were of chronic neuralgia; once only he obtained a radical cure by the section of the suborbital nerve; the three other times he could only give partial relief.

*Causes.* Twice the disease arose from a traumatic cause; in one, from a fall on the head, and was cured; in the other, from a piece of iron falling against the left suborbital nerve. Twice again it was caused by fright; in the first case the fits became less painful and frequent with time, and the patient died of apoplexy at the end of ten years; in the second case it occupied the right frontal branch, was terribly intense, and lasted eight years, when it yielded; but it reappeared in the left frontal with less intensity, lasted again two years, and disappeared spontaneously, leaving behind it violent frontal headach, which brought on mania. Neuralgiæ caused by terror appear to be exceedingly obstinate. Bellingeri has only once seen neuralgia commence at the period of cessation of the menses, and once in a man on suppression of hæmorrhoids; antiphlogistics and sedatives cured these cases. A rheumatic cause is one of the most frequent of neuralgia. It consists in the prolonged action of humid cold on the body, or sudden changes of temperature, or cold playing on the face. Of this he gives several instances, chiefly left suborbital neuralgia; it is very obstinate when originating in cold.

*Species of Neuralgia.* Bellingeri divides neuralgia of the face into inflammatory and irritative, or nervous. The inflammatory he subdivides into the sanguineous, phlogistic, and rheumatic. The sanguineous depends on a simple congestion of the encephalon or nervous trunks, and is owing to suppressed menses, hæmorrhoides, or other evacuations &c.: there is general plethora. The phlogistic is an actual phlogosis of the nervous trunk, or of its origin, consequent on a prolonged congestion, or on a traumatic cause; suppression of transpiration, repercussion of exanthemata, &c. induce it: a serous effusion may accompany it, and thicken the neurilema. It should be remembered, that most frequently neuralgiæ have at their commencement more or less of a phlogistic character, or of simple sanguineous congestion, and that then antiphlogistic means of varied power should always be employed. Traumatic neuralgia are in this case; but, on the other hand, the wounding cause often produces lesion of the same, requiring particular means. Bellingeri has seen neuralgia from wounds cured by antiphlogistic and sedative remedies when treated early, where, as in old cases of the same kind, these remedies only palliated. Rheumatic neuralgia is caused by cold and humidity: early, it requires antiphlogistics and sedatives; at a later period revulsives and diaphoretics are necessary: it is the most frequent kind, and also the most curable, in Bellingeri's hands.

Irritative neuralgia depends on a foreign body irritating the nerve, as caries, worms, tumors of the frontal or maxillary sinus, retrocession of exanthemata, or syphilis: in treatment the cause must be removed.

Purely nervous neuralgia is irregularly intermittent or periodically intermittent. Idiopathic neuralgia, essentially nervous from its commencement, is very rare: of this kind are those produced by mental causes: also, those that are sympathetic in their commencement, for subsequently the repeated attacks beget an actual phlogosis in the nervous trunk; to this kind, also, may be referred the inflammatory or irritative neuralgia, that persist after all inflammation has been subdued and all cause of irritation has been removed. When periodical intermittent neuralgia is purely nervous, it only requires quinine; but, depending on an inflammatory or rheumatic cause, it calls for antiphlogistics in the first instance. Almost invariably, when quinine is required, it is well to premise antiphlogistics, otherwise the disease is likely to be aggravated; for it is rarely the case that a neuralgia, even essentially nervous, is not accompanied at its commencement with inflammatory congestion.

*Treatment.* Regularly periodical neuralgia yields with certainty to quinine: reference, however, must be had to what has been said above on the possibility of an inflammatory state being allied with the pain; rheumatic neuralgia, properly treated, is converted into regular periodic, and treated accordingly. When neuralgia is phlogistic, it is sometimes necessary for some time to persist in bloodletting. In neuritis, besides bleeding, purgatives, and sedatives, as laurel-water and hyoscyamus, are to be used; twenty-eight cases of neuralgia were successfully treated in this manner by Bellingeri; he gives twenty or thirty grains in the day. Acetate of morphia is of little use taken internally; it is more effectually used in friction, with oil, on the pained part; or better still, on the denuded dermoid tissue. Three intense rheumatic neuralgiæ were in this manner cured, antiphlogistics being previously used. Bellingeri raises a blister, and sprinkles half a grain of the acetate on the surface every twenty-four hours: he saw two grains cause fainting, nausea, &c. When neuralgia is rheumatic after antiphlogistics, we should raise a blister on the arm of the corresponding side, and keep it open for a long time; some diaphoretic (kermes, with extract of aconitum,) hastens the cure. Traumatic neuralgiæ are to be treated antiphlogistically; if the nerve has been pricked or lacerated, or contused, and the pain resists all antiphlogistics, section and cauterization of the nerve, a little below the point of lesion, should be practised. Irritative neuralgia, which has for cause some product of inflammation, as serous infiltration, thickening of the neurilema, tumefaction of the nerve, &c. should be treated by friction with mercury or iodine, general or local. When owing to repelled eruptions, they should be recalled; when to syphilis, recourse must be had to mercury. Sedatives are more peculiarly fit in the essentially nervous neuralgia; if the patient is feeble or lymphatic, preparations of steel or quinine may be added, or should rather be premised. When owing to a mental affection, oxide of zinc, valerian, ammoniuret of copper, prussiate of iron, &c., may be used; but Bellingeri has never seen these means successful in old cases. Section and cauterization should be practised between the brain and the disorganized part of the nerve; two sections should be made, and the intervening portion removed, lest cicatrization should renew the connexion with the brain; this is better than the horribly painful cauterization of the extremities of the nerves. For the same reason Bellingeri rejects simple cauterization of the nerve, as proposed by Paletta, and consisting in the division of the nerve with a red-hot knife.—*Dr. Ryan's Journal.*

### TUMOURS OF THE NECK.

Two cases are narrated by Dr. N. R. Smith, in the Fourth Number of the *Baltimore Journal* in which he extirpated tumours of the neck. In one the termination was favourable.

"In the winter of 1830, I visited Mrs. Gilliland, aged about twenty-five years, the wife of a farmer living near Gettysburg, Pa., for the purpose of examining a large tumor situated in her throat. I found it to be located on the right of the thyroid cartilage, under the border of the sterno-cleido-mastoid, and having the omohyoid muscle strained directly across its centre. It was of course completely beneath the deep fascia, and was in immediate contact with the sheath of the great vessels. Its form was ovoidal, its size that of a large goose's egg, and it occupied nearly the whole space from the angle of the jaw to the clavicle, creating great deformity. Its long diameter corresponded to the length of the mastoid muscle. It was very firmly bound down by the mastoid muscle and fascia, and was moved with great difficulty beneath them. I first satisfied myself that it was no portion of the thyroid gland. I also convinced myself that it did not involve in disease the great vessels and nerves of the neck. That by its mechanical pressure it irritated these organs was sufficiently manifest, for there existed a train of symptoms evidently resulting from mechanical pressure on the pneumogastric nerve. The stomach was much impaired in its functions, her appetite being capricious, and food often occasioning much distress in the organ soon after being taken. She was also sometimes affected with nausea, diarrhœa, alternating with costiveness. There was also not a little embarrassment of respiration. She suffered severely with occasional pains in the head, on the side corresponding to the disease. The pulse also gave evidence of considerable constitutional irritation."

The patient recovered completely. In the other instance, Dr. Smith extirpated a similar tumour from the neck of a farmer, aged forty-two, who recovered, and remained in good health for about a year. The tumour then returned in the same spot, but it was now hard, knotted, closely adherent to the surrounding spots, and excessively painful. Dr. Smith determined to attempt to remove the tumour.

" Accordingly, in the presence of my friends, Drs. Shorb and Miller, and several of my pupils, I divided the integuments on the inner side of the cicatrix, and proceeded to explore its connexions. After cautiously dissecting it from the muscles, and separating its external connexions, I introduced the finger into the wound, and made gentle efforts to detach and separate the tumor from the vessels. While I was in the act of doing this I felt something give way to a gentle effort of the finger, as if some soft substance were ruptured by it, and instantly the wound, the table, and the floor was deluged with dark blood.

I immediately discovered that the internal jugular vein had become involved in the disease, and that its coats, having become soft and brittle, had been largely rent by even the slight traction which had been made upon them. The vessel appeared also to have become enlarged, and as by the struggles of the patient, the irregularity of breathing, and the action of the heart, the blood was pressed with great force from the cava into the jugular, the hemorrhage was truly appalling. Hemorrhage from the carotid, I am confident, could not have been so rapid. It bubbled so copiously from the wound, that in an instant, and before I could press my thumb into the bottom of the cavity, the floor was covered with blood, and the patient fell back inanimate, and as if dying. Respiration and circulation being thus suspended, I was perfectly aware that instant action, before any reaction should occur, was necessary, to save our patient from death on the operating-table. I opened the wound more freely with the knife. I removed the friable portions of the tumor, I exposed the vein, and found it torn open down close to its junction with the subclavian. I then seized an armed needle, which was at hand, and at the moment that the patient was reviving, and that blood began again to gush, I passed a strong ligature beneath the vessel, and secured it close behind the clavicle. This was done with some apprehension, lest I should include the pneumo-gastric nerve. After encircling the vessel, however, I satisfied myself that the nerve was not included, and immediately drew the knot. There was still a good deal of venous hemorrhage from the upper orifice of the wound, and an oozing of arterial blood from the remainder of the diseased medullary mass. It was manifest that the coats of the artery also were involved and converted into the peculiar structure of the disease, and that any further effort upon the latter would at once produce an arterial hemorrhage. I therefore at once closed the wound, and applied to it as the most efficient compress which could be used in that region, a soft sponge. The bleeding immediately ceased, and slight pulse returned in the extremities. The patient was carried to his bed; and, although he suffered greatly, and was much reduced, immediate dissolution was no longer threatened.

"I was obliged to leave my patient, to return to Baltimore, soon after the operation, but the conclusion of the case was related to me by Dr. Shorb, of Littlestown. He survived the operation about ten days, and then sunk, apparently from exhaustion. It is manifest therefore that he could not have perished from phlebitis, which by some is supposed almost necessary to arise from the application of ligatures to veins of such very large size, and in a diseased condition.

"Another fact is worthy of particular notice. At the moment that blood was gushing most copiously from the wound, when the patient was fainting, and the inspirations were unfrequent, deep and strong, I distinctly heard a bubbling of air as it was sucked into the vein. I apprehended at the moment that immediate death would be the consequence, but I am not aware that any particular morbid phenomenon resulted from it."

### A CASE OF CONGENITAL DEFICIENCY OF BOTH THE UPPER AND LOWER EXTREMITIES,

By J. F. E. Hardy, M.D., of Ashville, North Carolina.

The subject of this deformity is a young woman, aged about twenty years. She was born without either upper or lower extremities, the situation of which is merely occupied by small rounded projections. The stumps of the shoulders are remarkably small and short; those of the thigh are much larger, but are not more than two inches in length. I think her mother has had twelve children, but she is much larger than either of her brothers or sisters. She is, indeed, of a full and plump habit, and possesses a peculiarly lively disposition.

Her power of locomotion is remarkable. She can transport herself over the floor with considerable ease, which she does by submitting her body to a kind of rotatory motion alternately from right to left, and the contrary. By confining the handle of a broom between her chin and shoulder, she can sweep the floor with considerable dexterity. She can also sit erect, lean back or rock herself in a chair, as well as another person; and, when any thing is given her, she makes a sign for it to be placed upon her shoulder. If it be any solid article of food, she eats it from that situation.

Her hips and nates are remarkably full and large, and are almost square. Her breasts are also voluminous, and remarkably plump, presenting all the characters of the mammæ of a stout young female of her age. Her catamenial discharge is regular, and of the natural quantity.—*Baltimore Med. Journal.*

### A CASE OF POISONING BY FISH.

My attendance upon Mrs. H. a confidential servant, æt. 40, commenced Jan. 26, 1835. She was then labouring under slight pneumonia, slight indeed as far as regarded the prominence of the symptoms, but perhaps violent with reference to the scanty powers of resistance of an extremely feeble constitution. One very small bleeding, the application of leeches several times repeated, and two blisters, were among the chief remedies employed. She also took blue pill and squill, to the amount of 64 grains of the former, and 16 of the latter. But it is unnecessary to dilate upon the treatment of the case; let it suffice to state that every thing went on well till the 28th of February, with the exception of the pulse, which was rarely under 108, and more frequently 120. On the evening of the day just mentioned, I was suddenly called to my patient, and found her sitting up in bed, speechless, but with her eyes open, and apparently sensible; or rather with the vague consciousness of a person suffering under a "phantasma, or a hideous dream." She occasionally pointed to her ears and mouth, by which she seemed to signify that she could hear but not speak. The case, I will confess, was a perplexing one. Although I was aware that Mrs. H. had formerly had a paralytic stroke, I was not likely to fall into the error of supposing that another attack was at hand, or of employing the lancet to cut the knot which I could not untie; for the pulse kept up its usual rapidity, the pupil contracted when a candle was brought near the eye, and there was nothing like stertor or coma.

The bystanders assured me that Mrs. H. had taken nothing which could have disagreed with her. I began with giving her small quantities of Epsom salts, and meantime renewed my inquiries as to her dinner. The fish, her friends repeated, was quite sweet; on examining the remainder (part of a sole), I found it quite fetid. The mystery of the case seemed to be cleared up.

Twenty grains of sulphate of zinc were immediately procured and administered; but as eight or nine hours had elapsed since dinner, nothing but mucus and water was vomited. In about an hour, Mrs. H. recovered her consciousness, recognised me with surprise, asked if she had been ill, &c. &c. but soon relapsed. The recovery and relapse were repeated; she again recovered, and in rather more than two hours after my arrival, I left her tolerably composed, but confused and distressed by the consciousness of the dreamy state from which it had been so difficult to rouse her. She complained of her head having been oppressed, of having seen many lights, and so on. The numerous doses of sulphate of magnesia given during the evening were not without effect, in spite of the vomiting, for a stool was passed in the night, and was reported to be extremely offensive. Some days elapsed before the dimness of her sensations cleared up; and meantime a new source of irritation, a large abscess near the rectum, made its appearance, and in its turn caused delirium; so that, from the two attacks thus melting into one another, it was not easy to say how long it was before she recovered from the first. I ought not to omit to state that before, as well as after, the attack of Feb. 28, she took half a grain of the acetate of morphia nightly; but so far was this from producing any unpleasant effect, that the patient herself remarked to me, on the morning preceding the attack, that she had supposed that all sleeping-draughts affected the head, but that this did not. Her recovery has been perfect. My opinion, which I give with considerable diffidence, is that these remarkable symptoms were caused by the fish-poison, after it had reached the small intestines, and could no longer be recalled by an emetic.

*March* 27, 1835. E. A. DOMEIER.

### SYRUP OF RHUBARB STALKS.

M. Chevallier has given an account of his method of preparing a syrup from the Rheum Australe, in the Bulletin Géneral de Thérapeutique. A leaf was taken, weighing 416 grammes, and stripped of the green part, so as to leave the stalk and its ramifications, amounting to 320 grammes. This was bruised in a wooden mortar, and then pressed: the juice thus obtained was first passed through linen, to separate the coarsest particles, and after being suffered to settle for some days, was then filtered. The filtered juice weighed 266 grammes, and was made into a syrup in the following manner. The 266 grammes of filtered juice were put into a matrass, and 525 grammes of powdered white sugar being added, the matrass was heated in a water-bath, and the heat was kept up until the sugar was perfectly melted and converted into syrup, at the top of which was a white pellicle. The syrup was then passed through a sieve; it was transparent, but when cold, it was found that a white powdery precipitate had taken place. This was removed, and proved to be oxalate of lime, proceeding either from the juice, or the sugar, or both. The remaining syrup was clear, and of a fine amber colour. Its taste was slightly acid, and very pleasant, resembling that of the syrup made with rennet apples.

This syrup owes its agreeable acidity to the superoxalate of potash. M. Chevallier has since prepared it with the stalks of other varieties of rhubarb, the rheum compactum, undulatum, and almatum.

EFFECTS OF INDIGO IN SPASMODIC DISEASES.

Dr. Moritz Strahl gives the result of some experiments on this subject, in Gräfe and Walther's Journal, (Vol. xxi. Part I.) He first employed indigo in cases of confirmed epilepsy, beginning with a scruple three times a-day, and increasing the dose until half an ounce was taken daily. During the use of the remedy, the stools became blue, and the urine dark green. Ten epileptic patients, of whom four were under 15 years of age, and the rest from 20 to 30, took the indigo without intermission for eight weeks. In two cases only there was a temporary absence of the disease, but even in these it returned with as much violence as ever. The indigo seemed to have no effect on the system, with the exception of its dyeing the stools and urine, and causing a slight uneasiness of the stomach, which was probably merely a mechanical effect, depending on its bulk, as it occurred only from the largest doses, and generally disappeared in a few hours. These patients were all males. The author likewise administered indigo to four hysterical patients, one of whom was in extreme old age, and the others were girls aged 17, 21, and 24. The effects were singular enough. When the dose had been raised to two drachms a-day, all four were seized with violent nephritic pains, the urine was more intensely coloured than in men, and a tolerable quantity of fine indigo dust was to be seen at the bottom of the vessel. These violent renal symptoms lasted four days; and yielded at last to the continued use of an oily emulsion. In one case only was there a remission of the spasmodic symptoms after the disappearance of the nephritic attack, and that patient is now quite well, three months after the discontinuance of medicine. The power of the indigo over the uterus was sufficiently striking; for in two cases an amenorrhœa, with which the spasmodic symptoms were complicated, was entirely cured, though the spasms themselves did not cease when menstruation had returned. In two cases of St. Vitus's Dance, one in a boy of 12, and the other in a girl of 9, the indigo was administered for six weeks, but without benefit. Dr. Moritz Strahl concludes that indigo is inefficacious in spasmodic diseases, but possesses considerable power over the genital and uropoetic system.

BELLADONNA IN HOOPING-COUGH.

This remedy has been used with success by M. Guersent in two cases. The first patient was a girl aged five and a half, who had been labouring under hooping-cough for a fortnight. The dose was a grain of the alcoholic extract of belladonna daily, to begin with, and was gradually augmented to three grains. It did not produce any unpleasant symptom except dilatation of the pupils. Amendment rapidly took place, and in six days the cough became purely catarrhal, but the remedy was continued three days longer, to prevent all danger of a relapse.

The second patient was a girl aged seven. She had had a cough three months, and had hooped three weeks. In this case the belladonna cured the hooping in a few days; but being now omitted the hooping returned. The belladonna was resumed, and again cured the hooping, but caused enormous dilatation of the pupils, confused vision, and head-ache. The dose was diminished, and the disease did not return.—*Gazette des Hôpitaux.*

### ON THE USE OF CAMPHOR IN TYMPANITIS.

Dr. Tradini has narrated four cases of tympanitis in the Gazette Médicale. The first one, he says, he treated according to the doctrines of Sauvages, Morgagni, and Cullen. The patient was a woman about 60, of corpulent habit, who had always lived comfortably and enjoyed good health. The author applied a large blister to the abdomen, and ordered the following mixture.—℞ Aq. Menth.Pip.; Aq. Melissæ aa. ℥ii.; Æther. Sulph. ʒi.; Syrupi ℥i. M. sumat coch. ii. ampla ter in die. A temporary alleviation ensued, but nothing was of any permanent benefit, and the patient died in three months.

Case ii.—The patient, a man æt. 45, was first treated by the application of iced water and ice to the abdomen ; but as this was of no benefit, Dr. Tradini prescribed the following remedies:—R. Camph. pulv. gr. vi.; Extr. Cinch. officin. gummosi, gr. viii. M. ft. pilula; dentur tales æquales n°. viginti, sumat unam quarta q. horâ.—Frictions with hot flannel, and the use of flannel drawers and waistcoats were likewise ordered. Eighteen of the boluses completed the cure.

Case iii.—The patient was the famous improvisatore Philip Pistrucci, whom our author treated in London. He had previously been under the care of another physician, who had prescribed aperients, which had opened the bowels, but not relieved the abdominal distention. The same remedies were employed, except that each dose contained seven grains of camphor, and in a few days Pistrucci was cured.

Case iv.—The patient was a boy, æt. 9, who had been ill for a month, and kept his bed for a week. He was suffering from marasmus as well as tympanitis, and there was fluctuation in the abdomen. The prescriptions were—R. Camph. pulv. gr. ii.; Scillæ pulv. gr. ss.; Extr. Cinch. officin. gummosi, gr. ix. M. fiat pilula; dentur tales n°. 6, sumat i. omni tertiâ horâ ante nutrimentum. —R. Decocti graminis, ℥x.; sit pro potu.—The diet was to consist of broth and wine. These measures produced immediate relief, and in a month the child was quite well, with the exception of some glandular swellings in the neck.

### ON STAMMERING AND ITS CURE.

There is a paper on this subject, by Dr. Moritz Strahl, in Gräfe and Walther's Journal. The method of which he gives an account was brought to Europe from America, by a Mrs. Leigh. She entered into partnership with Dr. Malbouche at Brussels, from whom the secret was bought by the Belgian government. The system has likewise met with great applause in Prussia, and was designated by Dr. Zitterland, member of a commission appointed to inquire into its merits, one of the most important discoveries of our age. The Prussian medical board, too, sent persons acquainted with the method to the seminaries where schoolmasters are educated, in order to instruct these teachers how to cure any cases of stammering which might occur to them. Dr. Strahl learned the art from his sister, who had been under the care of a professed curer of stammering.

The whole art consists in the following rules: the stammerer is to press the tip of his tongue, as hard as he can, against the upper row of teeth, is to draw a deep breath every six minutes, and is to keep perfect silence for three days, during which, this pressing of the tongue and the deep inspirations are to be continued without intermission.

During the night small rolls of linen are placed under the tongue, in order to give it the required direction even during sleep. When the three days have expired, the patient is to read aloud slowly to his physician for an hour. During this exercise care is to be taken that the stammerer is never in want of breath, and he must therefore be made to stop frequently, and inspire deeply. The patient is to be admonished to keep the tip of the tongue floating when he speaks, and never to allow it to sink into the anterior cavity of the lower jaw.

It would hardly be believed that this most simple method of treatment could have such striking success as experience teaches us that it has, even in the worst cases. Whether it is that speech is made more easy by the mechanical direction of the tongue, which, after the three days of pressure, remains of itself in the floating direction, or whether it is from the moral influence exercised by the three days' silence and the mystery that hangs about the treatment; certain it is, that the result is immediate, and surprisingly favourable, but unfortunately it is not lasting.

Having thus given the method of cure, I will now, says Dr. Strahl, narrate the results of several cases.

Case I. Matilda F. the daughter of a person holding a respectable official situation at D., a slender blonde of 18, in perfectly good health, had suffered since her ninth year so severely from stammering, that she was obliged to confine all intercourse to the circle of her nearest relations, because conversation was so difficult to her, that after some violent preliminary stammering, she was no longer able to utter a syllable. She was well educated, quite familiar with the German and French classic authors, and though lively, of a gentle disposition. When she spoke to acquaintances her words flowed on for some time, until at last some word came, which she could not pronounce without the greatest difficulty; and when such a stumbling-block had once occurred, her confusion increased, and she stammered much more frequently, even in words which just before had been pronounced with the greatest facility. Neither in this nor in any other patient whom I have treated, have I perceived that any initial letter caused especial difficulty; there was hardly a word in the language which was not at one moment pronounced with ease, and a few minutes afterwards unutterable. In the patient of whom we are now treating, the stammering was particularly striking when she read aloud. She could not utter three words running without a convulsion of stuttering strongly affecting her facial muscles, and painfully embarrassing to her auditors. When her father introduced her to me I handed her a book, in order that I might judge of the degree of the defect from her manner of reading. Three lines cost her eight minutes, and caused a spasm which excited the liveliest compassion of the by-standers. It was remarkable that reading aloud produced stammering even before her friends, though she could converse with them tolerably well. The patient remained in my house, had a room to herself, and observed the rules detailed above for three days with the greatest attention. On the fourth day I made her read aloud, and was astonished to find that she could read two whole pages without the smallest stumbling. It is true that she did it very slowly, but she had been told to do so on the former occasion without any effect. She talked with such ease, that strangers who were accidentally present could not detect any defect in her utterance. In order to make matters sure, I continued the reading exercises for three days, and then began a new period of silence with its annexed conditions. After the lapse of this second period of three days she read aloud with great firmness, and it very seldom happened, that either in speaking or reading any difficulty occurred, and if it did, it was easily overcome. I announced this fortunate result to her father, requesting him to take her home; on his arrival, I had a party at which Goethe's Tasso was read, and my patient took the part of the Princess, which she read with such ease, and in so feeling and correct a style, that she enchanted all who were present. This was universally recognised as a radical cure.

Six weeks afterwards I went to D. where I had the mortification to hear from her father that the defect had returned in the severest form; that she was no longer able to read aloud at the family prayers as she had done for the first few weeks after her return, and that she stammered when talking to strangers. My patient was now sent for, and although our conversation was long and lively, she did not stammer once; she read aloud, and succeeded perfectly in that also. This favourable result was obviously due to the moral excitement caused by the presence of her physician. Her father concluded from this, that a longer residence in my house, and a repetition of the curative process, must be useful to her: but although she lived for six weeks more under my constant superintendence, and I subjected her to several kinds of exercises, I was no longer able to produce the favourable result which I had observed after the first three days; and I was obliged to dismiss my patient without being able to consider her as cured, though she was certainly much relieved. She had evidently gained in firmness of utterance, and even in reading aloud the spasms were but slight, though remarkable.

Case ii. Theodore L., a young man aged twenty-five, town-clerk of M., lively and of a sanguine temperament, but of small intellectual cultivation, stammered to such a degree that he was always laughed at by uneducated persons; his greatest annoyance, however, was the being obliged to read aloud the documents which it was his duty to draw up. He was three times subjected to the course described above; the amelioration was in his case also greatest after the first three days, but it did not progress by the repetitions of the treatment, and after four weeks' attempts the patient was dismissed, considerably relieved indeed, but by no means radically cured. He now reads out his papers with a very moderate effort, which in truth is so slight, that he holds himself for cured. Any passion, however, loses him the mastery of his speech, and a slight excitement of his feelings calls forth a strong stammering convulsion.

Case iii. Von N., the son of a rich Memnonite at D., was introduced to me by a letter from his father. I was sitting one afternoon at coffee with my family, when a well-dressed young man entered the room, and attempted to address me; in doing this he distorted the muscles of his face in such an extraordinary manner, that though we were all accustomed to stutterers, it required a forcible effort to smother our laughter. I immediately saw that this must be my new patient, and by addressing him accordingly, put an end to our mutual embarrassment. It turned out that Von N., who was a man of the gentlest and most tranquil disposition, stammered beyond any thing that I had ever heard before. He remained four weeks in my house, and to the astonishment of all my friends, as well as myself, was perfectly cured. The result was so permanent that even now, four years after his cure, he remains in the full and undisturbed possession of his organs of speech.

Case iv. Augustus D., æt. 12, the son of a peasant, stammered so shockingly, that the greatest attention did not enable a stranger to understand him. He was quite uneducated, because he could not receive any instruction, and even his reading was very so so. I attempted his cure, which, contrary to all expectation, was effected to a certain extent. After he had been three weeks in my house, he could often converse with my children for a quarter of an hour without stumbling, and could speak even with strangers with but little struggling, and in an unconstrained manner. It was obvious that an immense improvement had taken place. Six months afterwards I heard from his afflicted father that his stammering had returned with unweakened violence. Two years after he had been under my care I saw him again: he was a stout country lad, but the defect in his speech was as great as can well be imagined.

CASE V. Theodore F., æt. 14, the son of a clergyman, was a sprightly clever boy, who had stuttered from his infancy, and felt his defect the more painful, as he was conscious that it would be a great hindrance to him in the pursuit of the sciences which he wished to study. He submitted with readiness to the prescribed rules, but although he continued the exercises with patience, it was seven weeks before he was able to talk to strangers without embarrassment. At the expiration of a year I heard, from his father, that two months after the cure his son's stammering had returned, though in a less degree.

Dr. Strahl observes, with great justice, that Mrs. Leigh's method having radically cured one case and alleviated the others, is well worthy the attention of the scientific physician. He thinks that the principal advantage of this plan consists in its arresting the stammerer's attention by directing him to keep his tongue in a given position, and thus freeing him from the fear of stammering. But he is also of opinion, that the mechanical direction given to the tongue may facilitate speaking; and this supposition is confirmed by its effect in Case iv., where the patient, from the uncultivated state of his intellectual faculties, was not likely to be affected by a mere moral impression.

